# A case of idiopathic retroperitoneal fibrosis presenting as gallbladder carcinoma

**DOI:** 10.1186/s12893-021-01162-x

**Published:** 2021-03-25

**Authors:** Rui Li, Shengqi Fei, Yongfeng Lv, Xiangyu Kong, Weikun Mao

**Affiliations:** Department of Surgery, Changxing People’s Hospital, No. 66, Taihu Road, Changxing, Huzhou, 313100 Zhejiang China

**Keywords:** Retroperitoneal fibrosis, Gallbladder carcinoma, Laparotomy, Abdominal distension

## Abstract

**Background:**

Retroperitoneal fibrosis (RPF) is a rare disease with a poor prognosis characterized by systemic inflammation and fibroinflammatory tissue. Idiopathic RPF (IRPF) accounts for approximately two-thirds of RPF cases.

**Case presentation:**

A 56-year-old female patient with abdominal distension was admitted to Changxing Hospital. Laboratory tests revealed mild anemia and elevated CA125, while IgG4 and autoantibodies were within the normal ranges. Computed tomography (CT) revealed a gallbladder-occupying lesion, pancreatic cyst and retroperitoneal mass, which may have contributed to bilateral ureteral compression and hydronephrosis. The initial diagnosis was gallbladder carcinoma with lymph node metastasis. Then, abdominal adhesiolysis, cholecystectomy and partial hepatectomy were performed. Histologically, there were fibrosis and inflammation in the retroperitoneal tissue without any malignant cells in the retroperitoneal or gallbladder tissue. Finally, we confirmed the diagnosis of idiopathic retroperitoneal fibrosis, chronic cholecystitis and pancreatic cyst. The patient recovered well following the CT scan, in which dilatation of the bile duct was reduced, and effusion of the bilateral upper ureter was no longer significant.

**Conclusion:**

This atypical case illustrates that RPF can be combined with other biliary tract diseases. The coexistence of other diseases conceals the symptoms of RPF, which increases the difficulty of image identification. A high degree of suspicion is necessary for routine clinical work. As more cases are reported, further advances in the diagnosis and treatment of RPF can be expected.

## Background

Retroperitoneal fibrosis (RPF) is a rare disease with a poor prognosis characterized by systemic inflammation and fibroinflammatory tissue. Idiopathic RPF (IRPF) accounts for approximately two-thirds of RPF cases, and the remaining third are secondary to specific causes such as medications, neoplasms, radiotherapy, infection, surgery and malignancies [1, 2]. Unfortunately, there are no clear diagnostic criteria for RPF. Previous studies reported that imaging examinations are helpful for the diagnosis of RPF to some extent, while it is difficult to distinguish RPF from other diseases due to the lack of specific signs [1, 2]. Abdominal discomfort, which is a symptom of gallbladder carcinoma (GBC), can also indicate common biliary tract carcinoma, which has a high degree of malignancy and a poor prognosis [3]. There were an estimated 115,949 new cases and 84,695 cancer-related deaths worldwide due to GBC in 2020 [4]. GBC is characterized by easy local infiltration, lymph node metastasis, and local vascular infiltration [5]. Patients with early localized GBC are usually asymptomatic; however, even when symptoms are present, patients usually present with nonspecific symptoms such as abdominal pain, nausea or vomiting, anorexia, or weight loss [6]. In clinical practice, CT and MR are the workhorses for the diagnosis and staging of GBC. It is worth noting that GBC often presents as mass lesions, thickening of the gallbladder wall, and intraluminal masses [6]. In this paper, we describe a case of IRPF with chronic cholecystitis and pancreatic cyst that was initially diagnosed as GBC with retroperitoneal lymph node metastasis.

## Case presentation

**Fig. 1:**
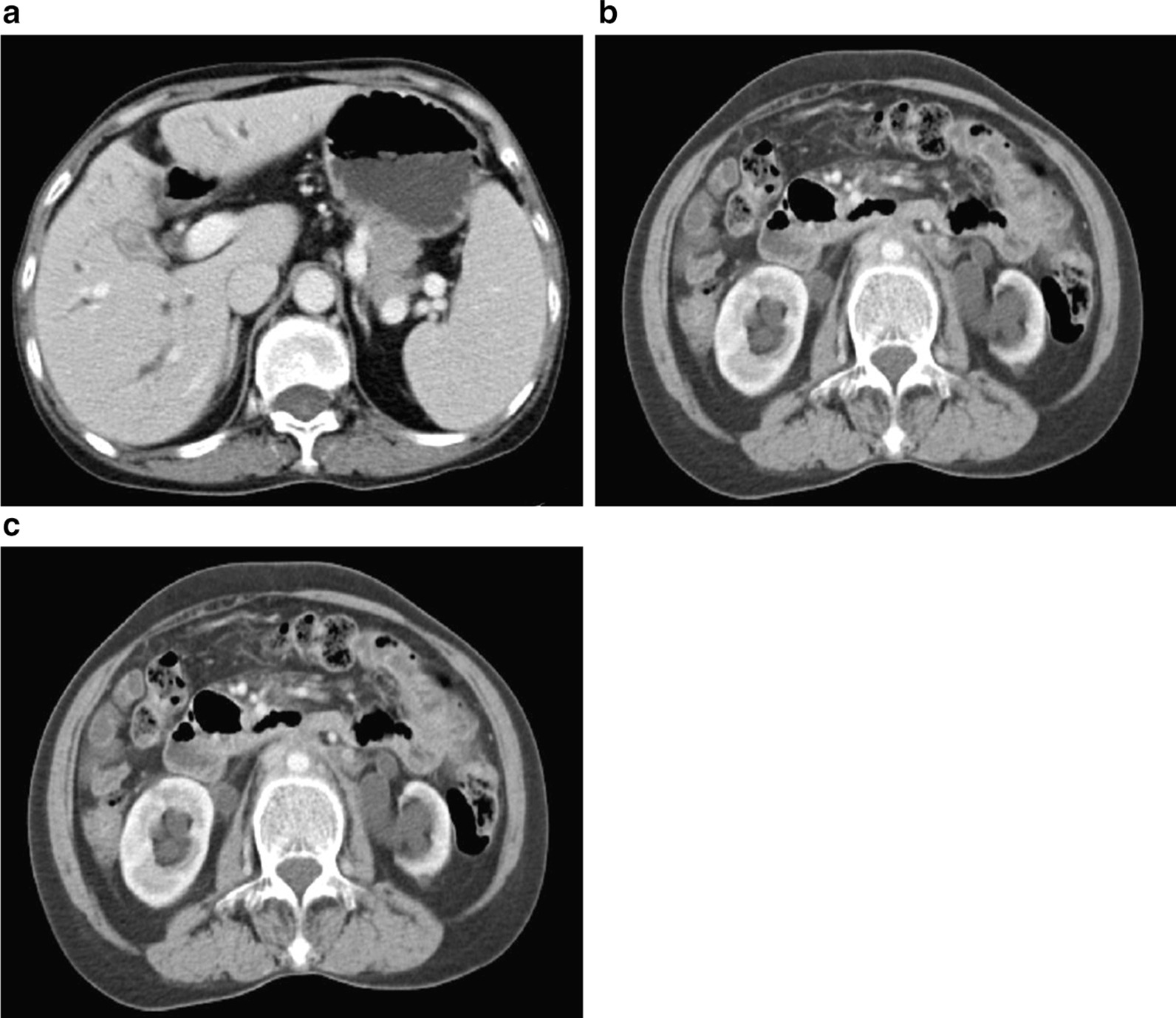
Characteristics of the abdominal CT scan. Abdominal computed tomography scan showing the gallbladder occupying and involving the hilar bile duct and dilatation of the proximal biliary system (**a**); posterior peritoneal mass occupying/involving the upper part of the bilateral ureter (**b**), and the change in the proximal urinary tract (**c**)

A 56-year-old woman with a history of rheumatism complained of right upper abdominal distension half a month prior. Abdominal distension was not accompanied by pain, exacerbated after meals and relieved after rest. Decreased urine output, nausea, vomiting, fever or chills were denied by the patient. Physical examination revealed the following: temperature, 37.2 °C; blood pressure, 123/70 mmHg; heart rate, 70 per minute; and respiratory rate, 18 per minute. There was no evidence of jaundice. Cardiac, pulmonary and neurologic abdominal examinations were all unremarkable. Dermatologic examination demonstrated pigmentation of both lower extremities. Laboratory tests showed elevated indirect bilirubin (15.2 μmol/L; RR, reference range: 1.7–10.2 μmol/L), decreased albumin (34.8 g/L, RR: 38–48 g/L), mild anemia with hemoglobin (97 g/L, RR: 110–150 g/L), and elevated CA125 (39.4 U/mL, RR: < 35 U/mL), while other tumor markers, creatinine, autoimmune and serum IgG4 were all within normal ranges. On the abdominal computed tomography (CT) scan, a rim of soft-tissue density in the retroperitoneum extended and encased the upper end of the bilateral ureter, causing bilateral hydronephrosis by ureteral obstruction (Fig. 1). In addition, an abnormally enhanced soft tissue density in the gallbladder was suspected to be involved with dilatation of the hilar bile duct and proximal biliary. A cystic density lesion in the tail of the pancreas was also noted. Given these findings, the diagnosis of GBC with retroperitoneal lymph node metastasis and pancreatic cyst was preliminarily considered, and immune-related diseases could not be completely ruled out.

**Fig. 2:**
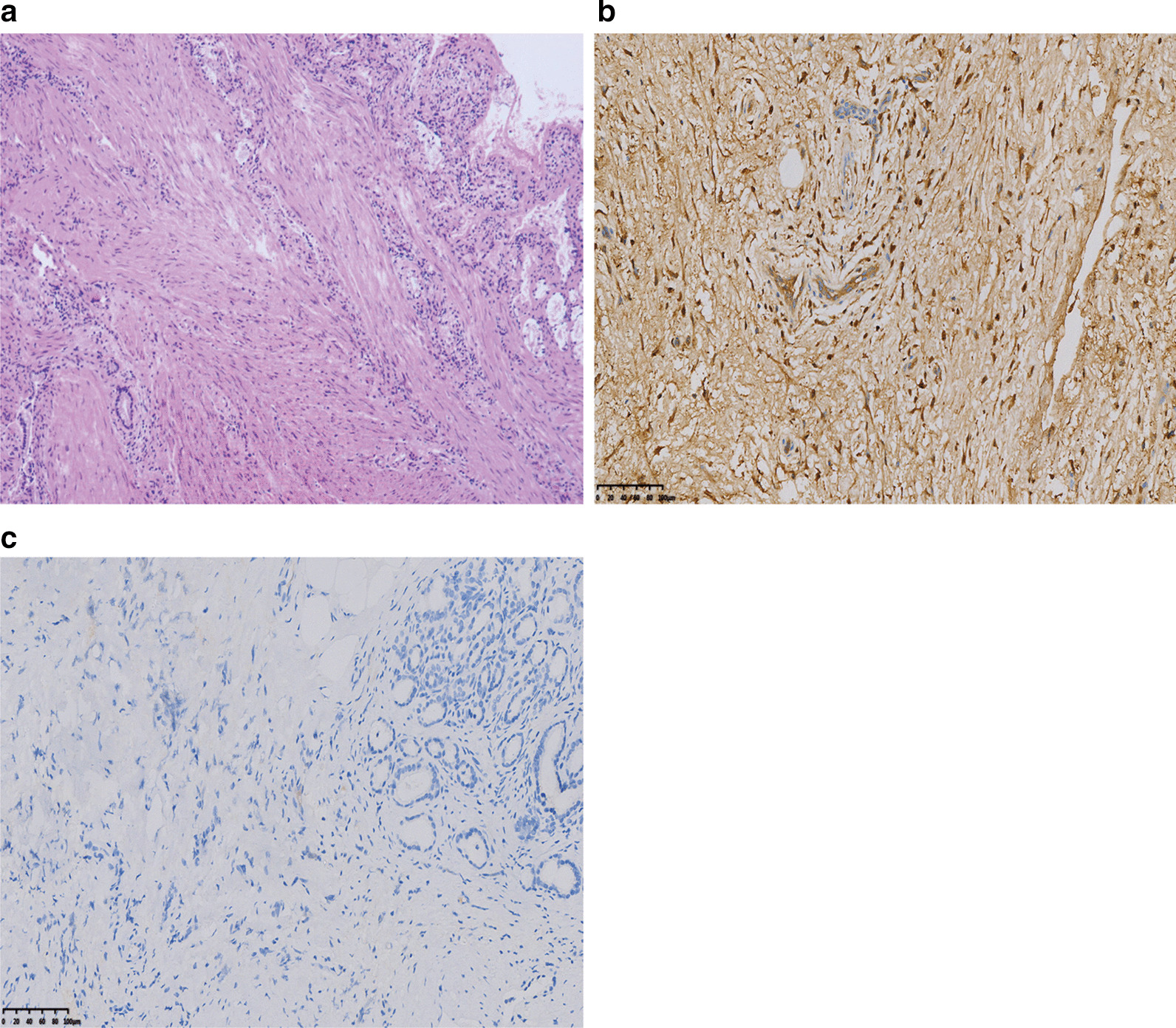
Histology of the retroperitoneal fibrous tissue. Pathology demonstrated dense fibrous tissue with lymphoid plasma cell infiltration (**a**). Immunohistochemical staining for IgG4 showed that the infiltrated plasma cells were IgG4 negative (> 200/high-power fields) (**b**, **c**)

**Fig. 3:**
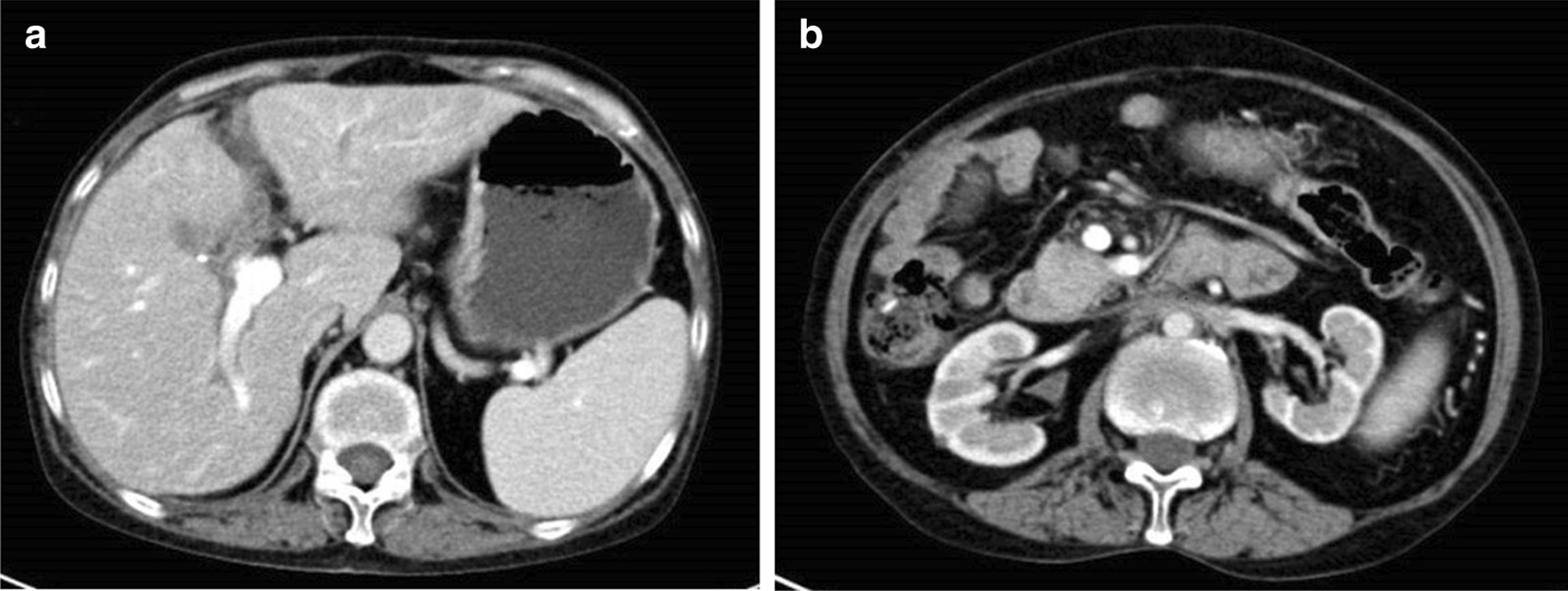
Repeat abdominal CT. **a** Dilatation of the intrahepatic and extrahepatic bile ducts. **b** Effusion of the bilateral upper ureter

Laparotomy was performed by abdominal adhesiolysis, cholecystectomy, partial hepatectomy, bilateral intraoperative transurethral ureteral stenting and cyst puncture. The retroperitoneum was filled with white and hard fibrous tissue. Pathologic examination showed mild chronic inflammation of fibrovascular tissue, diffuse proliferation of collagen fibers, interstitial edema, no increase in the proportion of plasma cells stained by IgG4, and no cell atypia, consistent with the pathological results of RPF (Fig. 2). The patient was discharged 10 days after the operation without any complications. Abdominal distension was gradually relieved after the postoperative oral administration of methylprednisolone (24 mg per day for 1 month). Four months after surgery, repeat abdominal CT showed that dilatation of the intrahepatic and extrahepatic bile ducts had improved compared with the previous scan (Fig. 3a), and effusion of the bilateral upper ureter was no longer significant (Fig. 3b).

## Discussion and conclusion

We experienced a case of a patient who complained of abdominal distension with chronic cholecystitis and pancreatic cyst who was initially diagnosed with GBC with retroperitoneal lymph node metastasis and ultimately diagnosed with IRPF.

Retroperitoneal fibrosis, also called Ormond’s disease, is a rare disorder with an estimated incidence of approximately 1.38 per 100,000 inhabitants [2]. Its incidence in men is slightly higher than that in women, with ratios ranging from 1:1 to 3:1 [7]. A few cases are secondary to specific causes, such as neoplasms, radiotherapy, infection, surgery, cancer and drugs. If no cause is identified, the remaining cases are classified as IRPF [1, 2]. RPF is marked by the presence of fibroinflammatory tissue. This specific tissue generally revolves around the infrarenal aorta, develops inferiorly toward the iliac bifurcation and probably encases adjacent structures such as the inferior vena cava and the ureters, finally leading to ureteral obstruction and hydronephrosis [1, 8]. There are three main hypotheses to clarify the pathogenesis of RPF, namely, atherosclerosis and autoimmune and IgG4-related diseases, but the exact mechanism of RPF remains to be further investigated [9].

Clinically, the most common chief complaint of RPF is abdominal or back pain, which may be dull and persistent and not aggravated by eating or exercise. Furthermore, less common complications include lower-extremity edema with possible deep venous thrombosis, constipation, scrotal swelling, varicocele and hydrocoele. When symptoms of renal failure appear, there is a high probability of ureteral obstruction [10, 11]. These symptoms are nonspecific and thus may not be helpful in the differential diagnosis. The clinical diagnosis of RPF depends on typical CT or magnetic resonance imaging (MRI) [12]. If there is a soft tissue shadow next to the abdominal aorta or iliac artery wrapping the surrounding tissue, such as the ureter or inferior vena cava, it often indicates RPF [13]. In this case, the patient mainly experiences painless right upper abdominal distension that is similar to the symptoms of chronic cholecystitis.

There were some similar typical cases published currently [14–16]; more details are provided in Table 1. Previous studies reported that abnormal laboratory findings included normochromic normocytic anemia, elevated inflammatory markers, abnormal renal function and positive autoimmune tests [10, 17]. Some cases were accompanied by elevated serum IgG4 [9, 18, 19]. Serum IgG4 may be elevated, but this finding is not useful for a differential diagnosis because IgG4 levels may also be high under other inflammatory conditions. Up to 40–50% of patients have been shown to have normal levels of IgG4 [20]. However, laboratory results are nonspecific and not sufficient to determine the diagnosis of RPF. This case showed only mild anemia, increased indirect bilirubin and a normal range of serum IgG. Increased tumor marker CA125 and decreased albumin levels suggested malignant disease.

**Table 1:** Characteristics of similar IRPF patients reported recently

Study	Age	Sex	Clinical symptoms	Histology	Initial impression
Chicoteau et al. [10]	72	Male	Abdominal pain	Inflammatory infiltrate	Dilatation of the intrahepatic bile ducts
Quante et al. [11]	55	Female	Jaundice	Chronic inflammatory fibrosis	Hilar cholangiocarcinoma
Fahd Khalil et al. [12]	60	Male	Jaundice	Acute inflammatory and infiltrative process	Hilar cholangiocarcinoma
Present study	56	Female	Abdominal distension	Fibrosis and inflammation	Gallbladder carcinoma

Concerning other laboratory examinations, a few other laboratory testing indicators should be considered, such as the erythrocyte sedimentation rate (ESR), C-reactive protein (CRP), anti-nuclear antibody (ANA) titer, and anti-neutrophilic cytoplasmic antibody (ANCA) titer [17]. In future clinical work, we should take these indexes into account to improve the accuracy of diagnosis.

Because imaging evidence is critical to the diagnosis of RPF, CT and MRI are considered to be of more importance than other devices [17]. Histological examination is required to confirm the diagnosis and to exclude the presence of potential malignancy or infection. In this case, the patient had an abnormally enhanced soft tissue density in the gallbladder and a retroperitoneal mass that increased the difficulty of diagnosis. The characteristic radiologic findings of chronic cholecystitis include continuity of the mucous layer and the homogenous contrast effect of the gallbladder wall on CT; however, it is challenging to differentiate chronic cholecystitis from gallbladder cancer in the case of localized thickening of the gallbladder wall. Subsequently, MRI and MRCP supported the previous diagnosis; thus, we first considered the diagnosis of a malignant tumor with lymph node metastasis. However, intraoperative findings and histological examinations confirmed that this was RPF without malignant lesions. Do we need to consider other diagnostic imaging techniques? Fludeoxyglucose (18F) positron emission tomography (18F-FDG PET) has been widely used in patients with cancer and inflammatory diseases and is now becoming more frequently used for the diagnosis of RPF.

The greatest limitation of our case was that a biopsy was not taken for the initial pathological diagnosis. In summary, this atypical case illustrates that RPF can be combined with other biliary tract diseases. The coexistence of other diseases conceals the symptoms of RPF, which increases the difficulty of image identification. A high degree of suspicion is necessary for routine clinical work. As more cases are reported, further advances in the diagnosis and treatment of RPF can be expected.

## Data Availability

All data during the study are included within the article.

## Abbreviations

RPF: Retroperitoneal fibrosis
IRPF: Idiopathic RPF
CT: Computed tomography

## References

[1] Urban ML, Palmisano A, Nicastro M, Corradi D, Buzio C, Vaglio A (2015). Idiopathic and secondary forms of retroperitoneal fibrosis: a diagnostic approach. Rev Med Intern.

[2] Tanaka R, Kameyama H, Shioi I, Ikeda Y, Hatakeyama S, Maruta T (2016). Laparoscopic right hemicolectomy for a patient with idiopathic retroperitoneal fibrosis: a case report. Asian J Endosc Surg.

[3] Huang YP, Liu K, Wang YX, Yang Y, Xiong L, Zhang ZJ (2021). Application and research progress of organoids in cholangiocarcinoma and gallbladder carcinoma. Am J Cancer Res.

[4] Sung H, Ferlay J, Siegel RL, Laversanne M, Soerjomataram I, Jemal A (2021). Global cancer statistics 2020: GLOBOCAN estimates of incidence and mortality worldwide for 36 cancers in 185 countries. CA Cancer J Clin.

[5] Liu F, Wu ZR, Hu HJ, Jin YW, Ma WJ, Wang JK (2020). Current status and future perspectives of minimally invasive surgery in gallbladder carcinoma. ANZ J Surg.

[6] Ganeshan D, Kambadakone A, Nikolaidis P, Subbiah V, Subbiah IM, Devine C (2021). Current update on gallbladder carcinoma. Abdom Radiol (NY).

[7] Scheel PJ, Feeley N (2013). Retroperitoneal fibrosis. Rheum Dis Clin N Am.

[8] Poduval G, Nathani P (2018). Idiopathic retroperitoneal fibrosis presenting as spastic paraparesis. Neurol India.

[9] Rossi GM, Rocco R, Accorsi Buttini E, Marvisi C, Vaglio A (2017). Idiopathic retroperitoneal fibrosis and its overlap with IgG4-related disease. Intern Emerg Med.

[10] Jadhav KK, Kumar V, Punatar CB, Joshi VS, Sagade SN (2017). Retroperitoneal fibrosis-clinical presentation and outcome analysis from urological perspective. Investig Clin Urol.

[11] Shiber S, Eliakim-Raz N, Yair M (2016). Retroperitoneal fibrosis: case series of five patients and review of the literature. Rev Bras Reumatol Engl Ed.

[12] Roussel E, Callemeyn J, Van Moerkercke W (2020). Standardized approach to idiopathic retroperitoneal fibrosis: a comprehensive review of the literature. Acta Clin Belg.

[13] Forestier A, Buob D, Mirault T, Puech P, Gnemmi V, Launay D (2018). No specific imaging pattern can help differentiate IgG4-related disease from idiopathic retroperitoneal fibrosis: 18 histologically proven cases. Clin Exp Rheumatol.

[14] Chicoteau J, Boudiaf M, Maillet M, Tran Minh ML, Lourenco N, Baudry C (2017). Extrinsic compression of the biliary tract due to idiopathic retroperitoneal fibrosis: MR imaging findings. Diagn Interv Imaging.

[15] Quante M, Appenrodt B, Randerath S, Wolff M, Fischer HP, Sauerbruch T (2009). Atypical Ormond's disease associated with bile duct stricture mimicking cholangiocarcinoma. Scand J Gastroenterol.

[16] Khalil F, Ouslim H, Mhanna T, Barki A (2015). Extensive primary retroperitoneal fibrosis (Ormond's disease) with common bile duct and ureteral obstruction: a rare case report. Int J Surg Case Rep.

[17] Tzou M, Gazeley DJ, Mason PJ (2014). Retroperitoneal fibrosis. Vasc Med.

[18] Konno S, Matsuno Y, Ichimiya S, Nishimura M, Kawakami Y (2019). Retroperitoneal fibrosis diagnosed as IgG4-related disease after 35 years. Intern Med.

[19] Hanly J, Kelly E, Jacobs C, Claridge A (2020). Non-renal idiopathic retroperitoneal fibrosis, a rare cause of abdominal symptoms. QJM.

[20] Fenaroli P, Maritati F, Vaglio A (2021). Into clinical practice: diagnosis and therapy of retroperitoneal fibrosis. Curr Rheumatol Rep.

